# Periodontal treatment prevents arthritis in mice and methotrexate ameliorates periodontal bone loss

**DOI:** 10.1038/s41598-019-44512-9

**Published:** 2019-05-31

**Authors:** Paul M. Lübcke, Meinolf N. B. Ebbers, Johann Volzke, Jana Bull, Susanne Kneitz, Robby Engelmann, Hermann Lang, Bernd Kreikemeyer, Brigitte Müller-Hilke

**Affiliations:** 1 Institute for Immunology, University Medical Center Rostock, Rostock, Germany; 2 Institute of Medical Microbiology, Virology and Hygiene, University Medical Center Rostock, Rostock, Germany; 3 Physiological Chemistry, Theodor Boveri Institute (Biocenter), University of Wuerzburg, Wuerzburg, Germany; 4 Department of Operative Dentistry and Periodontology, University Medical Center Rostock, Rostock, Germany

**Keywords:** Rheumatic diseases, Rheumatology

## Abstract

Recent studies indicate a causal relationship between the periodontal pathogen *P*. *gingivalis* and rheumatoid arthritis involving the production of autoantibodies against citrullinated peptides. We therefore postulated that therapeutic eradication *P*. *gingivalis* may ameliorate rheumatoid arthritis development and here turned to a mouse model in order to challenge our hypothesis. F1 (DBA/1 x B10.Q) mice were orally inoculated with *P*. *gingivalis* before collagen-induced arthritis was provoked. Chlorhexidine or metronidazole were orally administered either before or during the induction phase of arthritis and their effects on arthritis progression and alveolar bone loss were compared to intraperitoneally injected methotrexate. Arthritis incidence and severity were macroscopically scored and alveolar bone loss was evaluated via microcomputed tomography. Serum antibody titres against *P*. *gingivalis* were quantified by ELISA and microbial dysbiosis following oral inoculation was monitored in stool samples via microbiome analyses. Both, oral chlorhexidine and metronidazole reduced the incidence and ameliorated the severity of collagen-induced arthritis comparable to methotrexate. Likewise, all three therapies attenuated alveolar bone loss. Relative abundance of *Porphyromonadaceae* was increased after oral inoculation with *P*. *gingivalis* and decreased after treatment. This is the first study to describe beneficial effects of non-surgical periodontal treatment on collagen-induced arthritis in mice and suggests that mouthwash with chlorhexidine or metronidazole may also be beneficial for patients with rheumatoid arthritis and a coexisting periodontitis. Methotrexate ameliorated periodontitis in mice, further raising the possibility that methotrexate may also positively impact on the tooth supporting tissues of patients with rheumatoid arthritis.

## Introduction

Rheumatoid arthritis (RA) and periodontitis (PD) are frequent chronic inflammatory diseases. Approximately 1% of the human population worldwide suffers from RA, an autoimmune disease leading to destructive polyarthritis and extra-articular symptoms inflicting the heart, the lung as well as the renal and nervous systems^1,2^. RA is a complex disease with genetic and environmental factors contributing to its pathogenesis. However, the exact mechanisms and interactions leading to RA have not yet been elucidated^3–5^. PD on the other hand is an infectious disease that is characterized by the interplay of various pathobionts forming oral plaque and leading to the degradation of tooth-supporting tissues^6,7^. The estimated prevalence of chronic PD ranges between 1–20%^8,9^. Both, RA and PD therefore have substantial consequences on public health. Previous studies have shown that RA patients are more likely to suffer from PD compared to individuals without RA^10,11^. PD in turn is associated with a higher disease activity of RA patients^12,13^. These findings raised the question about the link between both disorders.

Epidemiological studies revealed that RA and PD share environmental risk factors, such as smoking and microbial dysbiosis^14,15^. Indeed, the fact that RA has been treated with antibiotics since the 1930s circumstantially supports the notion that bacterial infections may contribute to the pathogenesis of RA^16^. Additional cause-and-effect relationships were confirmed through clinical studies showing improved prognosis for RA-patients who were treated for PD^17^. Eventually, RA-specific antibodies against citrullinated peptide antigens (ACPA) that precede clinical symptoms of the joint disease turned out as the missing link between RA and PD^18,19^. Of note, one of the major pathobionts of PD, *Porphyromonas gingivalis*, was shown to citrullinate proteins via its bacterial peptidyl-arginine deiminase (PPAD)^20^. While citrullination in humans via human PADs is a physiological process, bacterial proteins modified via PPAD may function as foreign antigens that induce the generation of ACPAs^21^. Candidate antigens like bacterial enolase, that displays 80% homology to human enolase, may serve as initial targets for an immune response that via molecular mimicry and epitope spreading will eventually turn against self^22,23^. This scenario was supported by the finding that the ability of *P*. *gingivalis* to augment collagen-induced arthritis (CIA) in mice was dependent on the expression PPAD^24,25^. *Aggregatibacter actinomycetemcomitan*, which is another pathobiont associated with aggressive periodontitis in humans, was found to induce hypercitrullination in human neutrophils and was therefore considered yet another potential trigger for RA^26^. However, we ourselves previously demonstrated that oral gavage with *A*. *actinomycetemcomitans* led to significantly less alveolar bone loss in mice than *P*. *gingivalis*^20^. We therefore decided to focus on *P*. *gingivalis* for further studies and postulated that the specific therapy against *P*. *gingivalis* may ameliorate RA development. Since it is difficult to control the microbiome in a human setting, we stuck to the mouse model and subjected the animals to oral gavage with *P*. *gingivalis* in order to provoke PD^27^. We then induced collagen induced-arthritis (CIA), one of the most widely used animal models for RA^28,29^ and subsequently subjected the mice to various therapy regimen against either of these diseases.

PD-therapy traditionally centers on quadrant scaling and root planing^30^. Despite its good efficacy, some patients still suffer from ongoing PD afterwards. Adjunctive antimicrobial therapy with chlorhexidine gluconate or systemic antibiotics, especially metronidazole and amoxicillin reduce the risk of PD progression by preventing a recontamination from untreated pockets or other oral reservoirs^31,32^. Since mechanical therapy in mice is impossible to perform, we directly went for the subsequent antimicrobial therapy against *P*. *gingivalis* and compared orally administered metronidazole to chlorhexidine. We thus aimed to differentiate local and systemic effects on both, PD and CIA. Specific effects on CIA were compared to the treatment with methotrexate (MTX). MTX is not only the cornerstone of RA-therapy, but was previously confirmed to ameliorate CIA in mice^33,34^. In order to monitor microbial dysbiosis following oral inoculation, arthritis induction and subsequent bacterial eradication, we monitored the gut microbiome over the course of the experiment.

## Results

### Periodontal treatment prevents CIA in mice

**Figure 1:**
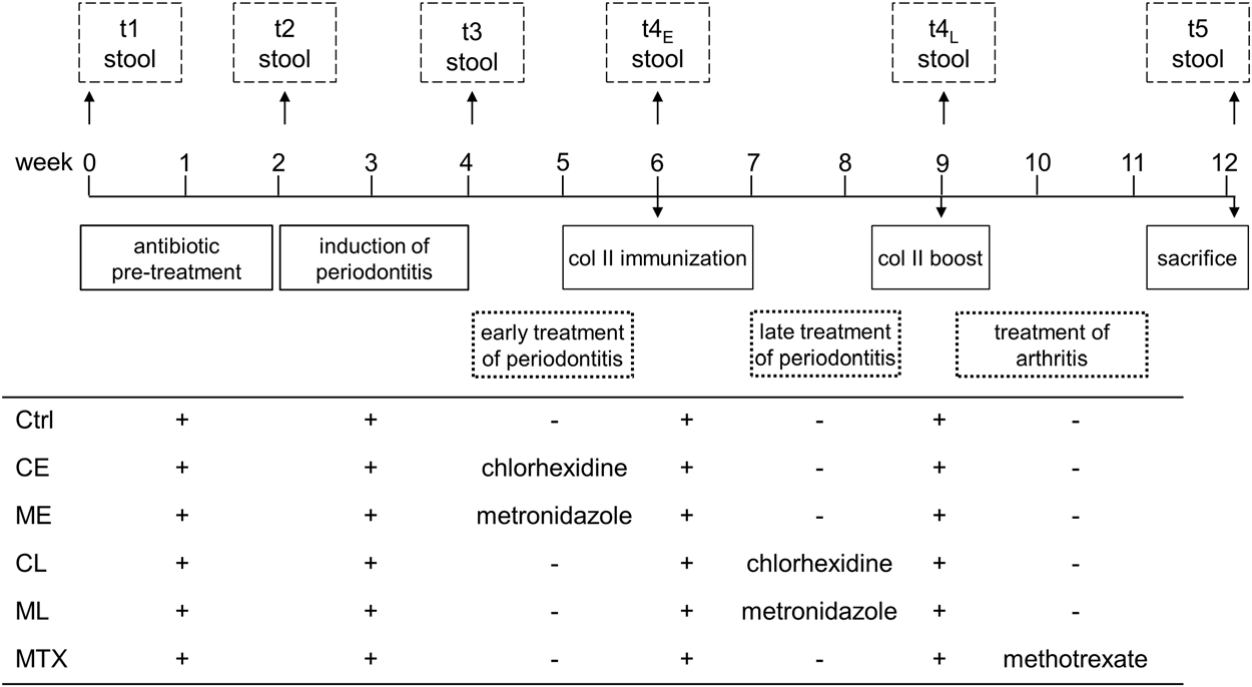
Schematic diagram of the study design. Mice were pretreated with antibiotics for 10 days, followed by a 3-day washout period before PD was induced via 8 consecutive oral inoculations with *Porphyromonas gingivalis*. After six weeks, mice were immunized with bovine collagen type II and boosted 3 weeks later. Four groups received oral applications of chlorhexidine or metronidazole either before (CE, ME) or during (CL, ML) arthritis induction. A fifth group received i.p. injections with methotrexate after the boost (MTX). The control group did not obtain any therapeutic intervention (Ctrl). Stool samples were taken at predefined time points (t1–t5). Mice were checked daily for visible signs of arthritis and were sacrificed at day 85. Time scale is given in weeks.

We here assessed the effect of a pharmacological treatment of periodontitis on the incidence and severity of arthritis. To that extent, periodontitis was first induced in DBA/1 J x B10.Q F1 mice via oral gavage with *P*. *gingivalis*. PD induction was then followed by either bactericidal (chlorhexidine) or antibiotic (metronidazole) treatment, either before (early) or during (late) induction of arthritis. The experimental scheme is depicted in Fig. 1 and shows another experimental group that received MTX for the direct treatment of arthritis. All groups were observed for identical time periods, independent of the treatment regimen.

**Figure 2:**
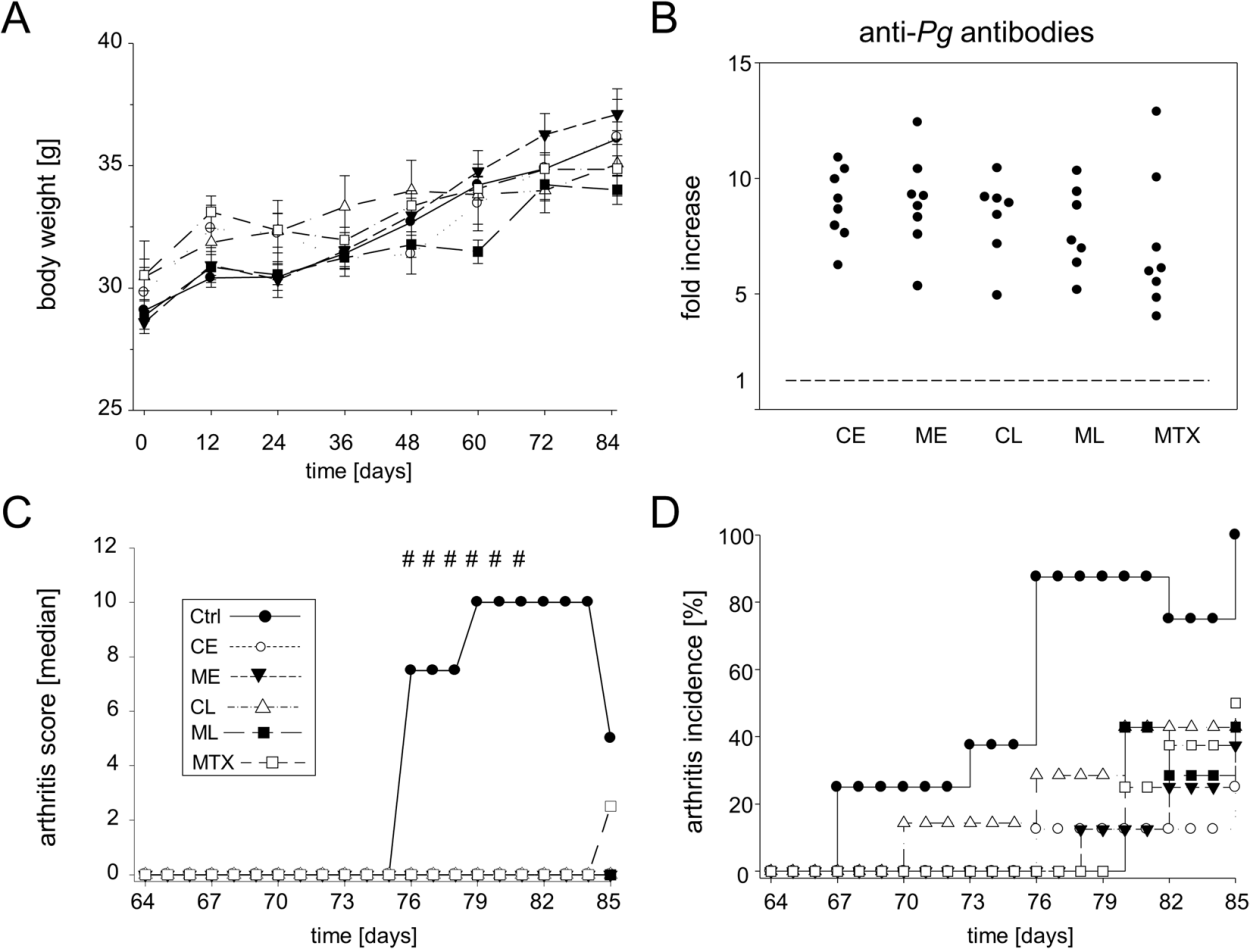
Non-surgical periodontal treatment ameliorates CIA in mice. (**A**) Weight gain over the experiment for the control group (Ctrl) and mice treated with either chlorhexidine, metronidazole (CE, ME, CL, ML) or methotrexate (MTX). (**B**) Fold increase in antibody titers comparing *P*. *gingivalis*-naïve mice with mice of our experimental groups. (**C**) displays the incidence of arthritis starting after the boost at day 64. Between groups differences in arthritis incidence were assessed via Log Rank tests and were statistically significant resulting in a P value of 0.00033. (**D**) Shows the median macroscopic arthritis score for each group. Hash keys denote significant between-group differences (p < 0.01) in arthritis scores as calculated via Kruskal-Wallis tests. Numbers of mice per group were Ctrl n = 8, CE n = 8, ME n = 8, CL n = 7, ML n = 7, MTX n = 8.

Mice were weighed and macroscopically scored for visible signs of arthritis every other day. The results in Fig. 2A show that the various experimental groups displayed comparable weight gains over the course of the observation period and thus developed physiologically. At the end of the observation period on day 85, mice were sacrificed and serum collected for quantitative analysis of antibody titers against *P*. *gingivalis*. All groups showed comparable and elevated anti-*P*. *gingivalis* titers which was considered proof of successful inoculation with the bacteria (Fig. 2B). Of note, 100% cumulative incidence of arthritis was observed at day 85 for the non-treated control group, only (Fig. 2C). All other groups displayed significant ameliorative effects of the various treatment regimen. The lowest incidence of 25% affected animals was found in the early chlorhexidine treated group that received treatment before immunization with collagen type II. Interestingly, MTX treatment had a slightly lower impact on the development of arthritis and resulted in a cumulative arthritis incidence of 50%. Between groups differences in arthritis incidence were assessed via Log Rank tests and were statistically significant resulting in a P value of 0.00033.

**Figure 3:**
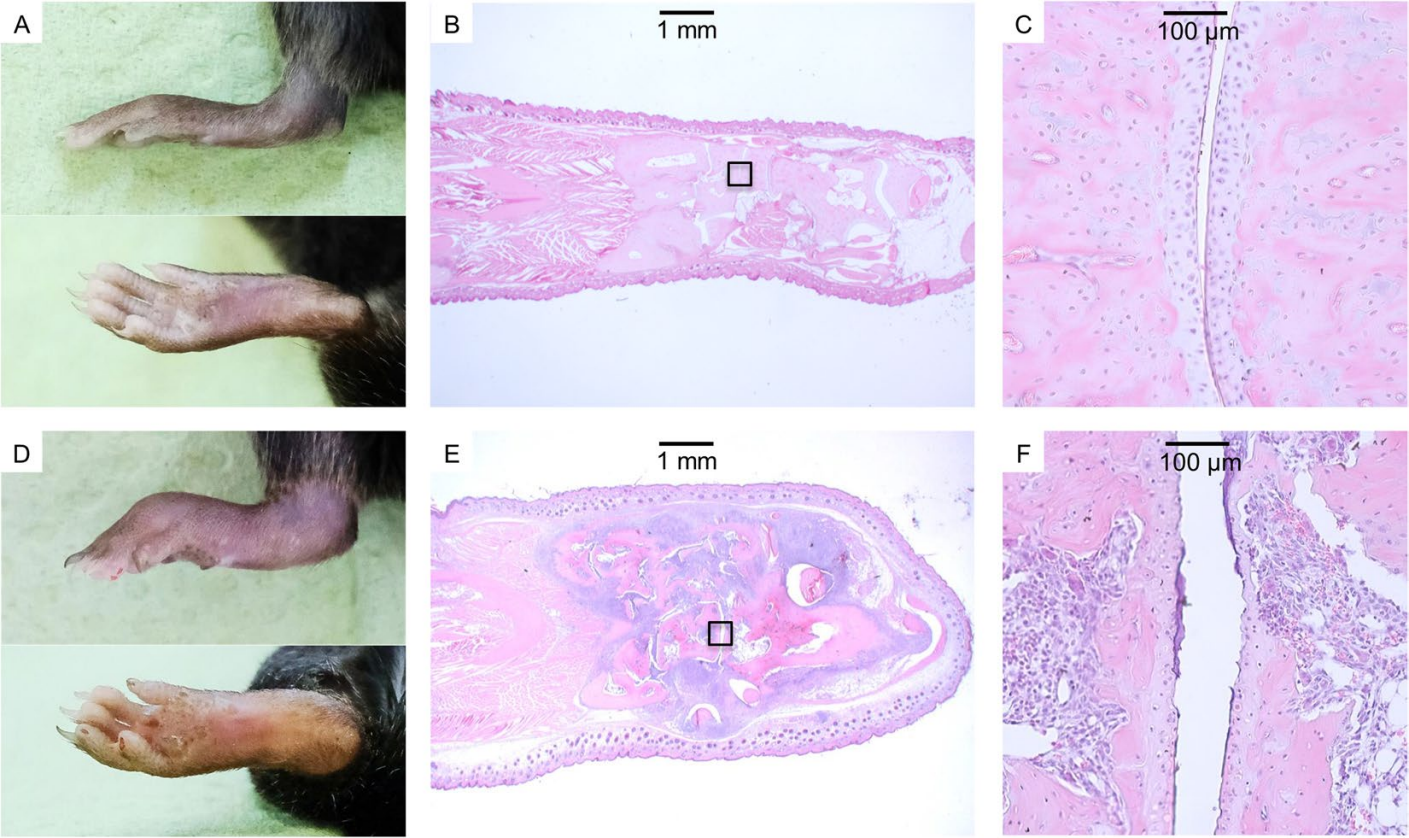
Macroscopic signs of arthritis were paralleled by overt histological inflammation. (**A**,**D**) Representative images of an unaffected and an affected (Score = 15) hind paw. (**B**,**C**) HE-stained thin-sections of the unaffected paw at 12.5x and 200x magnifications, respectively. (**E**,**F**) HE-stained thin-sections of the affected paw at 12.5x and 200x magnifications, showing massive leukocyte infiltration and cartilage destruction. Boxed areas in B and E denote the areas of magnification shown in C and F.

Arthritis scores were compared between all groups (Supplementary Appendix Fig. 2) and the medians between days 75 and 84 were up to 10 for the non-treated controls and 0 for all other groups (Fig. 2D). At the end of the observation period on day 85, median arthritis scores were 5 for the non-treated control, 2.5 for the MTX treated and 0 for all other groups. Mice without any macroscopic score remained inconspicuous during subsequent histological examination (Fig. 3A–C). Macroscopically overt arthritis was paralleled by histological signs of inflammation, cartilage destruction and bone erosion (Fig. 3D–F).

### RA-treatment ameliorates periodontitis in mice

**Figure 4:**
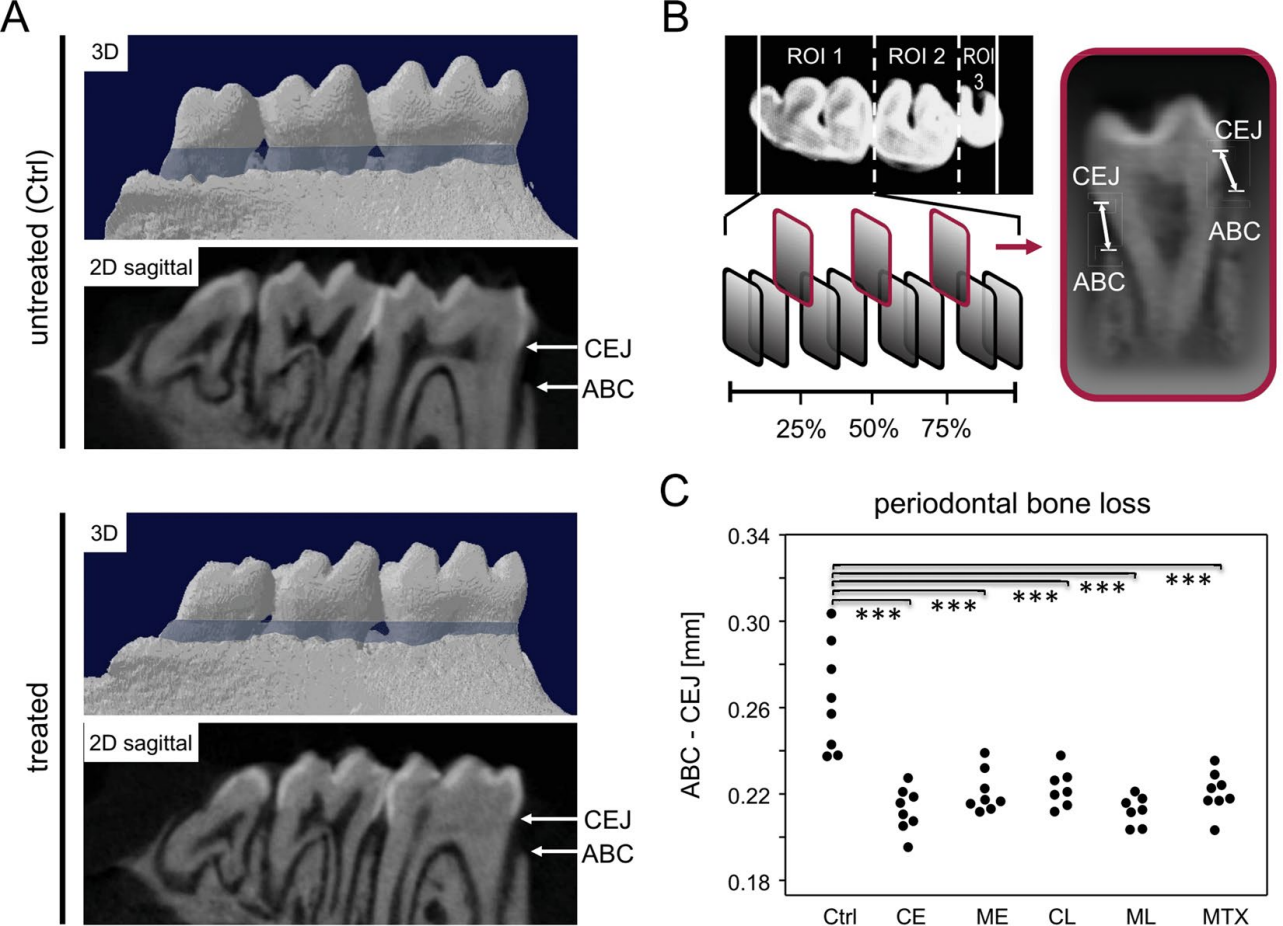
RA-treatment ameliorates periodontitis in mice. (**A**) Representative 2D and 3D images of the alveolar bone loss (blue area) found in untreated (Ctrl) and treated mice. Arrows indicate the cemento-enamel junction (CEJ) and the alveolar bone crest (ABC). (**B**) The upper left panel illustrates the axial plane of the right hemi-mandible and its three molars as well as the defined region of interest (ROI) for each tooth. The lower left panel indicates 3 geometrically distributed measurement planes per tooth, in which the bone loss was assessed. Right panel displays the measurement of the distance from CEJ to the ABC at the lingual and the buccal side of each tooth. (**B**) Shows the periodontal bone loss measured as an increased distance between the CEJ and ABC. The control group (Ctrl) received no therapeutic intervention. Numbers of mice per group were Ctrl n = 8, CE n = 8, ME n = 8, CL n = 7, ML n = 7, MTX n = 8. Asterisks denote statistically significant differences resulting from ANOVA. ***p < 0.001.

To quantify periodontitis, mandibles were imaged via µCT (Fig. 4A). We determined 3 geometrically distributed measurement planes per tooth in order to scale the distances between alveolar bone crest (ABC) and cemento-enamel junction (CEJ) (Fig. 4B).

The non-treated control mice displayed a significant 1.2-fold higher bone loss than the treated groups (Fig. 4C). Of note, intraperitoneal MTX injection turned out to be as protective against periodontal bone loss as orally administered chlorhexidine or metronidazole. There were no differences in bone loss between groups treated early or late.

### Orally administered *P*. *gingivalis* reversibly altered the gut microbiome

**Figure 5:**
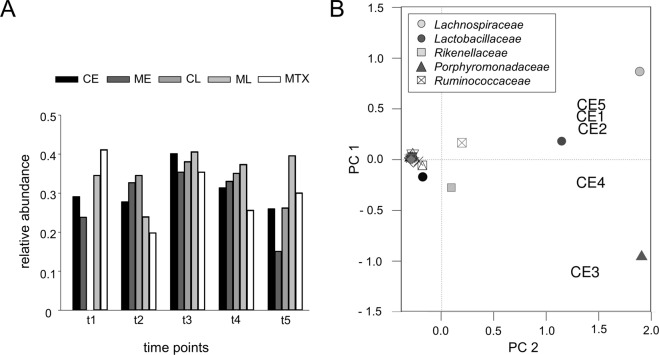
Orally administered *P*. *gingivalis* reversibly altered the gut microbiome. (**A**) Relative abundances of *Porphyromonadaceae* in the gut microbiome of mice orally treated with chlorhexidine (CE, CL), metronidazole (ME, ML) or methotrexate (MTX). Fresh stool was obtained t1) at the beginning of the observation period, t2) before and t3) after oral inoculation with *P*. *gingivalis*, t4) after pharmacological treatment and t5) at the end of the observation period. For the MTX-group, t4-samples were obtained before treatment. Analysis of CL at t1 was not done. (**B**) Principal components analysis plot shows the clustering of the most abundant bacterial families and the 5 samples of the early-treated chlorhexidine group (CE). Samples are numbered chronologically (CE1 = t1, CE2 = t2, CE3 = t3, CE4 = t4, CE5 = t5).

We next set out to assess experimentally provoked changes to the microbiome. We therefore collected fresh stool at five time points, t1: at the beginning of the observation period, t2: before and t3: after oral inoculation with *P*. *gingivalis*, t4: after pharmacological treatment and t5: at the end of the observation period. T4-samples for the MTX treated group were obtained before MTX application. The stool of four mice each was pooled and DNA was eluted. Sequencing of fecal samples generated a mean of 45,466 reads. At t1, the relative abundance of *Porphyromonadaceae* differed among the five groups. (Fig. 5A). As expected, there was a general trend towards an increase of *P*. *gingivalis* at t3 and a subsequent decrease after the individual treatments. The beta diversity analysis was performed using principal coordinates analysis (PCoA). Figure 5B shows an exemplary 2-dimensional PCoA plot presenting the most abundant bacterial families for the early treated chlorhexidine group. At t1 (CE1), the microbiome clustered between the *Lachnospiraceae* and *Lactobacillaceae* family. Antibiotics in the drinking water had no major effect on the microbial diversity at t2 (CE2) while oral inoculation with *P*. *gingivalis* led to a measurable microbiome shift towards the *Porphyromonadaceae* family at t3 (CE3). However, after chlorhexidine treatment, the microbial composition changed back towards the *Lachnospiraceae* and *Lactobacillaceae* families at t4 (CE4) and almost reached its initial diversity at t5 (CE5). Similar changes have also been detected for all other groups.

## Discussion

We initially postulated that antimicrobial therapy after *P*. *gingivalis* induced periodontitis would ameliorate subsequent CIA development. Our hypothesis indeed proved successful as both, metronidazole and chlorhexidine reduce the incidence and severity of CIA. Importantly, the outcome was comparable to MTX treatment, which has previously been described to ameliorate CIA in mice^34^.

For RA, non-surgical periodontal therapy (NSPT) has repeatedly been shown to reduce the DAS28 in patients^35^. To our knowledge, we here describe for the first time an effect of NSPT on CIA in mice. Even though CIA only serves as a model for RA and results cannot directly be transferred to the human condition, our findings support a link between inflammation of the gingiva and the joints and allow for further investigation of the causal relationship between reduced periodontal inflammation, oral eradication of *P*. *gingivalis*, and the benefits of NSPT.

*P*. *gingivalis* in mice has been described to enhance both, alveolar and articular bone resorption^36–39^. We therefore postulated, that therapies aiming to eradicate *P*. *gingivalis* would attenuate bone loss. After oral application of antimicrobial substances we thus performed microcomputed tomography in order to quantify the tooth supporting tissues. We here show that indeed, pharmacological treatment of *P*. *gingivalis* ameliorated alveolar bone loss. To our surprise, this effect was independent of the start of therapy. At the end of our observation period, mice treated at the early time point showed similar results compared to mice treated at the later one. However, we can rule out that alveolar bone loss commenced after performing the late treatment as we have previously shown that an increased ABC-CEJ distance can already be detected at 5 weeks after the oral inoculation with *P*. *gingivalis* with a further increase evident after another three weeks^20^. We cannot explain the absence of differences in bone loss between mice treated early and those treated later. We would tentatively like to speculate that rodent dentition may have the potential to recover after eradication of the bone resorbing stimulus. We are aware though that further experiments are required in order to verify this speculation.

In our experimental set up, MTX and oral bactericidal and antibiotic treatments were comparable in their amelioration of alveolar bone loss. Of note, MTX has previously been shown to efficiently decrease serum levels of receptor activator of NF-ϰB ligand (RANKL) in RA-patients^40,41^. As *P*. *gingivalis* was shown to stimulate alveolar bone loss by inducing RANKL expression, we hypothesize that the beneficial effect of MTX on alveolar bone loss may be mediated via RANKL^42,43^. However, to validate this hypothesis it would be necessary to monitor RANKL-levels over the course of our experiments. Unfortunately, we only collected serum samples at the end of the experiments. Despite this limitation, we here present an ameliorating effect of MTX on *P*. *gingivalis* induced PD in mice and this is novel. Further studies need to address an impact of MTX-treatment on the tooth supporting tissue in RA-patients.

We finally set out to investigate the mucosal colonization with *P*. *gingivalis* and the effect of pharmacological therapy on the microbiome. As opposed to human patients where the oral microbiome can easily be assessed via saliva, plaque or sulcus fluid from periodontal pockets, these direct samples cannot be retrieved from life mice. We therefore used two proxies. First we analyzed the sera of our mice for the presence of antibodies against *P*. *gingivalis* in order to verify successful inoculation. Secondly, we collected stool samples to quantify the mucosal colonization at various time points. The latter analysis relied on the previous observation that orally administered *P*. *gingivalis* will survive the gastric passage and can therefore colonize the gut^44^.

We indeed detected elevated anti-*P*. *gingivalis* antibody titers for all groups that were orally inoculated with *P*. *gingivalis*. Furthermore, oral inoculation with *P*. *gingivalis* led to increased relative abundances of various *Porphyromonadaceae* within the gut microbiome. However, this shift was transient and reversed after therapy. This reversal could simply mean that the oral inoculation with *P*. *gingivalis* generated a bacterial reservoir that fed into the gut until pharmacological treatment led to an eradication of the bacteria. On the other hand, we also observed a decline in *Porphyromonadaceae* abundance after MTX therapy. We therefore believe that swallowed *P*. *gingivalis* could only temporarily settle in the gut and were over time displaced by the resident bacterial flora. This latter thesis is supported by the continuous decline of *Porphyromonadaceae* between t4 and t5. Moreover, CIA has previously been shown to induce changes to the gut microbiota and these changes were evident before the onset of arthritis^45^. We therefore cannot differentiate which of the changes to the intestinal microbiome were caused by CIA, by pharmacological treatment or by the interactions with commensal bacteria. Investigating this interplay will require further studies and altered experimental designs.

In summary, we here demonstrated for the first time, that mouthwash with chlorhexidine or oral therapy with metronidazole effectively reduces incidence and severity of CIA in mice pre-infected with *P*. *gingivalis*. These findings suggest that NSPT may also prove beneficial for RA-patients with coexisting periodontitis. Moreover, the observation that MTX ameliorated periodontal disease in mice raises the question if MTX also impacts positively on the tooth supporting tissue in RA-patients.

## Materials and Methods

### Mice

DBA/1 and B10.Q mice were purchased from Harlan Winkelmann (Borchen, Germany). Male (DBA/1 x B10.Q) F1 mice for all experiments were then bred in the animal care facility under specific pathogen free conditions. Only male mice were used, since female sex hormones were shown to suppress CIA in mice^46^. They were kept in a climate controlled environment with a 12-hour light/dark cycle, in a stocking density of 2–4 mice per cage. Food and water were given *ad libitum*. Mice were entered into the experiments at the age of 8 and 10 weeks. Throughout the experiment, mice were weighed every other day. For all oral procedures, including the induction and therapy of periodontitis, mice were anesthetized with intraperitoneal injections of 0.75 mg Esketamin (100 mg/ml, bela-pharm, Vechta, Germany)/0.05 mg Xylazin (20 mg/ml, Bayer AG, Leverkusen, Germany) per 10 g of body weight. After the oral administrations, mice were placed underneath a heat lamp for one hour without access to food or water. The experiments were approved by the local state’s animal care committee (Landesamt für Landwirtschaft, Lebensmittelsicherheit und Fischerei Mecklenburg-Vorpommern) 7221.3-1.1-052/14. All steps were executed in strict accordance with the guidelines for animal experiments and all efforts were made to minimize suffering.

### Bacterial strains

*Porphyromonas gingivalis* W83 strain was provided from the Institute of Medical Microbiology, Virology and Hygiene, University Medical Center Rostock, Rostock, Germany. The bacteria were grown overnight to mid-logarithmic phase under anaerobic atmosphere (10% CO_2_, 10% H_2_, 80% N_2_). The bacteria were then centrifuged, washed with PBS and resuspended in DMEM medium to a cell number of 2 × 10^9^ cells per 50 µl. Aliquots were stored at −80 °C until immediately before the oral inoculation. Frozen bacterial aliquots were regularly thawed and the CFU were counted to confirm continued viability.

### Induction of periodontitis

The induction of periodontitis was performed as described previously by Marchesan *et al*.^27^. To enable the colonization with *P*. *gingivalis*, mice first received a 10-day antibiotic therapy. Drinking water containing 2% of antibiotics (Cotrim K - ratiopharm 240 mg/5 ml, Ratiopharm, Ulm, Germany) was supplied *ad libitum*, followed by a 3-day washout period. Starting at day 14, oral inoculation was performed by applying 2 × 10^9^ cfu suspended in 50 µl PBS containing 2% sodium carboxymethylcellulose (Sigma-Aldrich, St. Louis, MO, USA). The procedure was repeated seven times every other day.

### Induction of collagen induced arthritis

For the induction of collagen induced arthritis (CIA) we followed Brand *et al*. with slight modifications^28,29^. All Mice were primarily immunized on day 42. To that extent, they were placed in a mouse restrainer and subcutaneously injected with 140 µg bovine type II collagen (mdbioscience, Egg b. Zürich, Switzerland) in 0.1 M acetic acid, emulsified in an equal volume of complete Freund’s adjuvant (CFA, Becton, Dickinson and Company, Franklin Lakes, NJ, USA) at both sides of the tail base. On day 64 all mice received a boost, containing 140 µg bovine type II collagen in 0.1 M acetic acid emulsified in an equal volume of incomplete Freund’s adjuvant (IFA).

### Non-surgical periodontal treatment

Mice were treated with oral applications of either 50 µl chlorhexidine digluconate solution 0.2% (Engelhard, Niederdorfelden, Germany) or 50 µl metronidazole gel 0.75% (Galderma, Düsseldorf, Germany) for six times, every second day. Two groups received an early therapy starting before the immunization at day 30 (CE_arly_/ME_arly_). Another two groups received late treatment starting at day 50 (CL_ate_/ML_ate_).

### Methotrexate treatment

For the treatment of CIA, we followed Lange *et al*.^34^. One group (MTX) received 8 intraperitoneal injections of 2.5 mg/kg methotrexate in NaCl 0.9% in a volume of 200 µl. The procedure was repeated every other day, starting at day 66.

### Assessment of alveolar bone loss

Mandibles were first fixed in 4% paraformaldehyde (PFA) for 7 days and then stored in 0.9% NaCl. Three-dimensional microcomputed tomography was performed using a SkyScan 1076 micro-CT scanner (Bruker, Billerica, MA, USA). For a reproducible evaluation, 18 measuring points were defined for each right hemimandible, as described previously^20^. In short, after three-dimensional alignment, 3 geometrically distributed sites were determined for the lingual and buccal side of each tooth. Distances from the cementoenamel junction (CEJ) to the alveolar bone crest (ABC) were then measured in millimeters and in parallel for all groups.

### Macroscopic scoring of arthritis and histological examination

In order to investigate the onset and severity of arthritis, mice were monitored every other day following immunization. Macroscopic signs of arthritis including swelling and erythema were rated with 5 points each for an affected paw or wrist joint, respectively and with 1 point for each affected digit. A maximum score of 15 points per paw and 60 points per mouse could thus be reached (Supplementary Appendix Fig. 1). Absence of any macroscopic sign of arthritis was scored as 0 points. All animals showing macroscopic signs of joint inflammation were considered diseased and were therefore evaluated positive for disease incidence.

At day 85, all paws were collected and fixed in formalin 4% for two weeks. Paws were then washed, decalcified using Usedecalc (Medite, Burgdorf, Germany) and embedded into paraffin. Tissue slices were stained with hematoxylin and eosin (HE). Images were obtained using 12.5x and 200x magnification (Axioplan 2, Zeiss, Oberkochen, Germany).

### Antibody analysis

About 1.5 ml blood derived from the orbital vein plexus was centrifuged and the serum was stored at −20 °C. The measuring of IgG antibody levels against *P*. *gingivalis* was carried out as previously described by Gemmell *et al*.^47^, with few modifications. For coating *P*. *gingivalis* proteins onto the plates, 2 × 10^9^ cfu were resuspended in 600 µL CO_3_^2−^/HCO_3_^−^ buffer (pH 9.4) containing protease inhibitor cocktail (Roche/Sigma Nr: 04693159001) and 1 mM EDTA, before homogenization at 6000 rpm, 3 times for 30 s using Precellys 24 (Stretton Scientific, Stretton, UK). After performing a Bradford-Assay for protein content determination, 100 µL of a 1 µg/mL protein solution was coated overnight onto MediSorp ELISA plates (Thermo Fisher Scientific, Waltham, MA, USA). Plates were washed with PBS-Tween 20 (0.05%) and blocked with 2% bovine serum albumin (Sigma-Aldrich, St. Louis, MO, USA). Mouse serum was applied at dilutions of 1:200. After 1.5 h at RT plates were washed 3 times and incubated with detection antibody at a dilution of 1:1000 for 1 h. For color reaction, 100 µL TMB substrate (Biolegend, San Diego, CA, USA) was added. Optical density was determined at an absorbance of 450 nm using an automated plate reader (Anthos htIII; Anthos Labtec Instruments, Salzburg, Austria).

### Stool DNA extraction, preparation of 16S rRNA gene sequencing libraries, sequencing run and data analysis

At day 1, 12, 30 and day 85, fresh stool from 4 mice per group was collected and stored at −80 °C. A fifth sample was collected after the completed therapy, or, for the MTX treated group, before MTX application. Stool was homogenized via Fastprep-24 (MP Biomedicals, Santa Ana, CA, USA) with 6 m/s for 1 min using the ZR-96 BashingBead Lysis Tubes (Zymo Research, Irvine, CA, USA). DNA was then eluted using the ZymoBIOMICS DNA Miniprep Kit (Zymo Research, Irvine, CA, USA). Routinely, the concentration of purified DNA was measured by Qubit and Nanodrop protocols and the amplicon PCR was started with DNA templates using a concentration of 5 ng/µl in 10 mM Tris pH 8.5. The applied primers targeted the V3/V4 region of the 16S rRNA encoding gene and were initially described by Herlemann *et al*.^48^. The PCR resulted in amplicon sizes of roughly 450 bp. All further steps in library preparation were performed according to the Illumina “16S Metagenomic Sequencing Library Preparation” protocol.

Briefly, PCR clean-up, Index PCR, PCR clean-up 2, library quantification, normalization and pooling were performed according to the above referred manual. Bioanalyzer DNA 1000 chips (Agilent Technologies) and Qubit kits (Thermo Fischer Scientific) were applied for quantity and quality controls of each individual sample library and the final library pool. Ten percent PhiX control was spiked into the final pool. 4 pM of the final library pool was subjected to one individual sequencing run using a 500 cycle V2 chemistry kit on an Illumina MiSeq machine. During the run roughly 1,000 (K/mm2) clusters were sequenced, generating ca. 15 million reads passing filter specs. Over 75% of the sequencing and index reads were found with a Qscore ≥30. All raw data fastq files were recovered from the machine and were used for sequence data analyses. Sequence data analyses were performed via quality filtering (permitted length: 290–295 bp, no ambiguous bases allowed) and merging of duplicated sequences. Alignment to the reference database (https://www.mothur.org/wiki/Silva_reference_files#Release_128) was done using Mothur^49^. Only OTUs with total abundance >=3 were considered. Sequences from archaea, chloroplasts eukaryota and mitochondria were removed. For descriptive community analysis and PCA plots, the CRAN package ‘vegan’ was used (https://cran.r-project.org/web/packages/vegan/index.html). Similarity was calculated as Jaccard index, as a measure for dissimilarity Bray-Curtis has been used.

### Statistical analyses

Data were analyzed for Gaussian distribution via Shapiro-Wilk test. Mann-Whitney tests were performed to compare two groups, for more groups a Kruskal-Wallis tests and ANOVA were used. Differences in incidences were compared using a Log Rank (Mantel Cox) test. Analyses were performed using Sigma Plot (Version 13.0, Systat Software, Erkrath, Germany) or SPSS (Version 22, IBM, Armonk, NY, USA). P values smaller than 0.05 were considered statistically significant.

## Supplementary information

scoring of arthritis
