# Supplementary material for: Periodontal treatment prevents arthritis in mice and methotrexate ameliorates periodontal bone loss
Source: Sci Rep. 2019 May 31;9:8128. doi: 10.1038/s41598-019-44512-9 (PMC6544621; doi:10.1038/s41598-019-44512-9)
Supplement: Supplementary file 1 — scoring of arthritis [file 41598_2019_44512_MOESM1_ESM.pdf]

Periodontal treatment prevents arthritis in mice and methotrexate ameliorates periodontal bone loss

- SUPPLEMENTARY INFORMATION -

Paul M. Lübcke, Meinolf N.B. Ebbers, Johann Volzke (MSc), Jana Bull, Susanne Kneitz (PhD), Robby Engelmann (PhD), Hermann Lang (MD), Bernd Kreikemeyer (PhD), Brigitte Müller-Hilke (PhD)

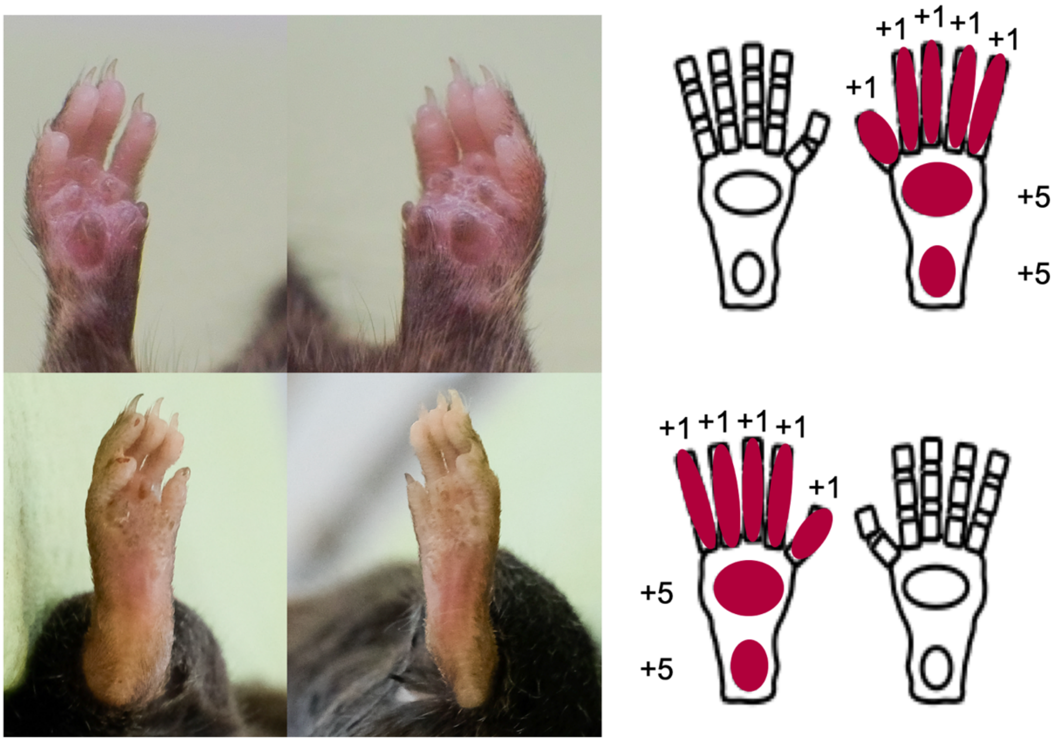

Supplemental Fig. 1 Scoring was performed according to the following scheme: 0 = no signs of arthritis, +1 = erythema and/or swelling for each affected digit, +5 = erythema and/or swelling for each affected paw area, +5 = erythema and/or swelling for each wrist joint. In this example both, the left front paw and the right hind paw were completely swollen and resulted in a total score of 30. The maximum possible score were 60 if all 4 paws were completely swollen.

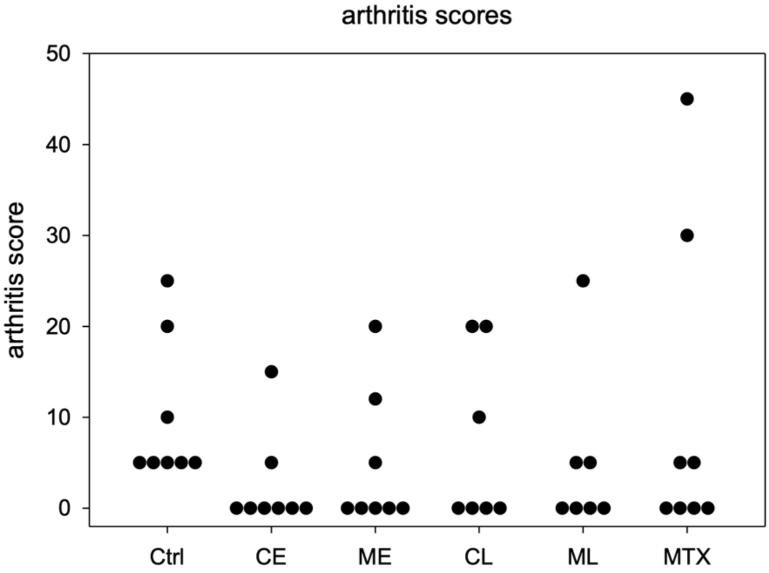

Supplemental Fig. 2 Arthritis scores for each mouse at the end of the experimental period. Macroscopic arthritis scores for the control group (Ctrl) and mice treated with either chlorhexidine, metronidazole (CE, ME, CL, ML) or methotrexate (MTX) at day 85. Numbers of mice per group were Ctrl n= 8, CE n= 8, ME n= 8, CL n= 7, ML n= 7, MTX n= 8.
